# The influence of an educational internet-based intervention in the beliefs and attitudes of primary care professionals on non-specific chronic low back pain: study protocol of a mixed methods approach

**DOI:** 10.1186/s12875-019-0919-6

**Published:** 2019-02-21

**Authors:** Ester García-Martínez, Jorge Soler-González, Francesc Rubí-Carnacea, Beatriz García-Martínez, Carolina Climent-Sanz, Joan Blanco-Blanco, Fran Valenzuela-Pascual

**Affiliations:** 1 Faculty of Nursing and Physiotherapy, University of Lleida, Lleida, Spain; 2 Grup d’Estudis Societat, Salut, Educació i Cultura, University of Lleida, Lleida, Spain; 3 Faculty of Medicine, University of Lleida, Lleida, Spain; 4 Institut Català de la Salut, Lleida, Spain; 5 Grup de Recerca de Cures en Salut, Institut de Recerca Biomèdica, Lleida, Spain

**Keywords:** Low Back pain, Chronic pain, Primary health care, Health personnel, Education professional, Educational technology, Pain neurophysiology, Gamification

## Abstract

**Background:**

Personal convictions in referral to pain cause misbeliefs in health professionals, which can influence patients who suffer from non-specific chronic low back pain. Likewise, health professionals’ beliefs affect their advice and attitudes towards patients’ treatment, becoming a possible cause of greater disability. The development of educational interventions based on the best scientific evidence in neurophysiology of pain could be a way to provide information and advice to primary care health professionals to change their cognition towards chronic non-specific low back pain. The use of Information and Communication Technologies allows the development of web sites, which might be one of the effective resources to modify misbeliefs and attitudes, in relation to the origin and meaning of non-specific chronic low back pain, of primary care professionals and that may modify their attitudes in patients’ treatment.

**Methods:**

The aim of this project is to identify misbeliefs and attitudes of primary care physicians and nurses about chronic non-specific low back pain to develop a web-based educational tool using different educational formats and gamification techniques. This study has a mixed-method sequential exploratory design. The participants are medical and nursing staff working in primary care centers in the city of Lleida, Spain. For the qualitative phase of this study, the authors will use personal semi-structured interviews. For the quantitative phase the authors will use an experimental study design. Subjects will be randomly allocated using a simple random sample technique. The intervention group will have access to the web site where they will find information related to non-specific chronic low back pain, based on the information obtained in the qualitative phase. The control group will have access to a video explaining the clinical practice guidelines on low back pain.

**Discussion:**

This study has been designed to explore and modify the beliefs and attitudes about chronic low back pain of physicians and nurses working in primary care settings, using a web-based educational tool with different educational formats and gamification techniques. The aim of the educational intervention is to change their knowledge about the origin and meaning of pain, with the result of reducing their misbeliefs and attitudes of fear avoidance.

**Trial registration:**

ClinicalTrials.gov Identifier: NCT02962817. Date of registration: 11/09/2016.

**Electronic supplementary material:**

The online version of this article (10.1186/s12875-019-0919-6) contains supplementary material, which is available to authorized users.

## Background

Low back pain (LBP) is a common musculoskeletal disorder and one of the main reasons why patients attend primary care (PC) centers, which has considerably high socioeconomic impact [1–7]. It is estimated that 60–90% of the adult population will experience LBP at some point in their lives and that between 5 and 10% will develop chronic low back pain (CLBP), defined by the International Association for the Study of Pain (IASP) as the pain that is lasting more than 12 weeks [1, 2, 8, 9]. The prevalence of CLBP increases linearly from the third decade of life to 60 years of age, being more frequent in women [7, 8]. In the context of PC, 80–90% of CLBP cases are considered of non-specific origin, since it is not possible to identify a specific cause that can be associated to the origin of pain [1, 2, 4].

Despite the high prevalence of pain in the population, as well as the burden and impact it poses to the individual, socio-family context and health systems, the IASP concluded that the time dedicated to pain in the education of health professionals (HP) is not enough to achieve effective pain management [10–13]. HP, as a result of their personal beliefs towards pain, often develop misbeliefs that have an impact on clinical approach of patients who suffer from non-specific chronic low back pain (NCLBP) [14]. A systematic review published in 2012 [15] concluded that HP’ beliefs and attitudes toward LBP have a direct influence on patients’ beliefs. They also affect the advice provided to their patients with NCLBP [3, 4, 15]. In addition, HP’ beliefs can alter patient’s interpretation of pain and cause misbeliefs and avoidance behaviors [16]. Several authors rely on the fear-avoidance model to explain the chronic pain process [14, 17, 18]. Patients make a catastrophic interpretation of the situation; they show fear towards physical activity and the development of possible injuries, exacerbating their pain [14, 17, 18]. In fact, fear towards pain is associated with avoidance behaviors, which have a negative impact on NCLBP, which it is strongly associated with disability, physical inactivity, increased drug dependence and overuse of medical services [14]. These avoidance behaviors in patients are reinforced by HP’ strong fear-avoidance beliefs, as they are more likely not to recommend or avoid physical and / or occupational activity and to prescribe passive treatments, sometimes resulting in an increased disability [14]. On the other hand, there is evidence that fear-avoidance beliefs in patients with NCLBP are reduced when they are treated by HP with less misbeliefs, despite the fact they advise patients to go on with their daily activities and occupational activities along with more active treatments, which correlates with a greater degree of functionality [14, 19]. Due to the immediate consequences of fear-avoidance beliefs in relation to health and well being of patients suffering from NCLBP, it may be good for the health system to understand them and to act upon them [14].

The study published by Darlow et al. [15] justifies the use of educational interventions to change the cognitive factors of HP involved in the treatment of NCLBP patients [15]. Several public health interventions have shown that HP’ re-education is effective in changing their cognition towards NCLBP and, consequently, changing their misbeliefs and attitudes towards treatment that may be reflected in those patients [20, 21]. The Societal Impact of Pain [22], a platform supported by the European Federation of IASP Chapters, urges governments and health institutions in the European Union to enhance education in knowledge, prevention, diagnosis and treatment of pain among HP. At present, HP who work in PC centers have clinical practice guidelines (CPG) in order to transfer knowledge to specific recommendations, assisting them in making individual clinical decisions and unifying therapeutic criteria, with the aim of promoting higher quality and welfare equity [23–26]. In Spain, the effectiveness of CPG in PC to change clinical practice is moderated [24]. In 2004, the Catalan Health Institute promoted a CPG for low back spine pathology in adults [27]. These guidelines include teaching material and advice for patients. However, they lack a neurophysiological explanation of the procedures involved in pain [27]. In their systematic review and meta-analysis, Clarke et al. [28] suggest pain neurophysiology (NPP) as a promising educational strategy towards pain and physical, social, and psychological function in patients suffering from NCLBP. It has also been shown to have positive effects on pain perception, disability and catastrophism in patients with chronic conditions [29, 30]. This educational strategy is based on the description of the central and peripheral processing mechanisms of the nociceptive signal and the explanation of how this transmission is modulated by brain processing and influenced by psychosocial factors [16, 31]. The results obtained so far suggest that an educational intervention based on NPP can help HP to conceptualize pain and to modify attitudes towards the treatment of chronic pain in PC [32].

The Information and Communication Technologies (ICT) in PC are a basic tool for patients’ and HP’ empowerment, improving their resolution capacity, efficiency and, consequently, attention quality [33]. Internet is one of the resources that has emerged with ICT development and has changed the way of accessing scientific information. Internet is a great source of information for health care management and an excellent channel of communication for HP. However, the influence it can have in professional practice and, more specifically, in the relationship between HP and patients is not clear [34]. It has been shown that web sites are capable of changing and improving the knowledge of chronic patients, in addition to having a positive impact on their attitudes and behavior [16]. This suggests that web sites could be an effective tool to influence the beliefs and attitudes of HP who work in PC.

Up to now, there is little literature on HP’ misbeliefs and attitudes towards the treatment and management of patients who suffer from NCLBP in PC [17]. Furthering these beliefs and attitudes will provide a greater understanding of the current pain explanatory methods used, as well as their possible impact on NCLBP treatment. HP should be aware of the relevance of these beliefs towards NCLBP approach, due to the fact that the omission of beliefs may be responsible for an increase in the degree of disability in patients.

As a consequence of the exposed previously and based on the results obtained by our research team [35], the objective of this study is to explore the HP’ misbeliefs and attitudes towards the treatment and management of NCLBP in PC to develop a web-based educational tool, based on the NPP.

The hypotheses being tested by our study are:

1. The beliefs and attitudes of fear-avoidance of primary care professionals are held by the lack of knowledge about the origin and meaning of pain.
2. The neurophysiology of pain as an educational intervention using a web site for primary care professionals will change their knowledge about the origin and meaning of pain, with the result of reducing their misbeliefs and attitudes of fear avoidance.

## Methods/design

### Objectives

There are specific objectives for each phase of the study.

- Phase 1 – Qualitative (QUAL): To identify the misbeliefs and attitudes of primary care professionals towards the treatment of chronic non-specific low back pain in relation to the origin and meaning of pain.
- Phase 2 - Connecting procedure: To build and develop, using a web site, a biopsychosocial educational intervention based on the results obtained in the qualitative phase.
- Phase 3 – Quantitative (QUAN):
  - ○ Primary outcome. To evaluate the effect of a biopsychosocial educational intervention about fear-avoidance beliefs related to non-specific chronic low back pain, using a web site for primary care health professionals.
  - ○ Secondary outcome. To evaluate the effect of a biopsychosocial educational intervention about the knowledge of pain neurophysiology, using a web site for primary care health professionals.

### Study design

To answer the research question a mixed-method sequential exploratory design will be used. This design consists of two stages, where the results of the methodology used in the first phase of the study, in this case QUAL, contribute to the development of the second QUAN phase [36]. In this project, both phases should have the same relevance for the development of an educational tool for PC professionals. The use of a mixed methodology design is justified in this protocol because the integration of both (QUAL and QUAN) methodologies occurs when the data from the QUAL phase contributes to the construction of the educational tool [16].

The process to be followed is:

- QUAL: Semi-structured personal interviews.
- QUAN: An experimental study design in which a sample of specialized physicians in family and community care and nurses working in PC health centers, will be randomly assigned to the experimental group and the control group.

### Eligibility criteria

The study population will consist of medical or nursing staff of both genders, working in PC centers in the city of Lleida, Spain.

Inclusion criteria:

- Professionals registered in the official college of medicine, specialized in family and community medicine or professionals registered in the official college of nursing.
- Professionals working in any of the PC centers of the city of Lleida.
- Accept and sign the informed consent form.

### Subjects

The recruitment process will be performed independently in each phase of the study, although the inclusion criteria will be common. To start the recruitment process, the first author will do a presentation of the project in each of the PC centers in the city of Lleida, addressed to the medical and nursing staff. Professionals will be invited to take part in the study and will be provided with a telephone number and an email address through which they can contact the first author.

- QUAL: Subjects who agree to be part of this phase of the study and who meet the inclusion criteria, will be contacted by the first author to agree on the semi-structured personal interview date. The interviews will take place in the same PC centers during the working day of the professionals to facilitate their participation.
- QUAN: The recruitment process will start after the end of phase 2. The QUAN phase will consist in a biopsychosocial educational intervention using a web site. The first author will meet individually with HP to ensure that they meet the inclusion criteria and inform them of the study conditions and provide them with the informed consent form. Also, the first author will answer any questions or concerns that may arise and will instruct them on how to access the web site to start their participation in the study.

### Sample size

- QUAL: The sample size will be determined by a convenience sampling method to achieve a representative number of HP. To ensure the discursive significance of the study results, the sample will be heterogeneous including doctors and nurses of different genders and age groups. The “snowball” strategy will be used to broaden the interviewed subjects. The final number of subjects will depend on the saturation of the information [37].
- QUAN: The sample size measurement is based on the study published by Domenech et al. [38] where the results obtained from the Health Care Providers Pain and Impairment Relationship Scale (HC-PAIRS) have been used as reference. Taking into account this study and other similar studies performed in general practitioners and physiotherapists [39–42], a standard deviation of 9 will be considered for the calculation of statistical power. A variation in the HC-PAIRS score of 4.5 (half the standard deviation) will be considered clinically significant. Accepting an alpha risk of 0.05 and a statistical power of 80% to reject the null hypothesis, using a bilateral test, the minimum number of participants will be 63 HP in each group. We assume that the dropout rate in the study will be 20%.

### Randomization

Subjects will be randomly allocated to either the intervention or control group using a simple randomization method. The subjects’ randomization will be performed by an external researcher from the Polytechnic School of the University of Lleida with a simple randomization technique using the STATS® program [43]. In order to ensure allocation concealment, the external investigator will provide codes created without a specific order in opaque envelopes that will constitute the randomized passwords to access the web site. The first author will deliver the envelopes with the passwords once the HP have signed the consent form. This will ensure the blinding of the evaluators in relation to the results. Due to the nature of the intervention, it will not be possible to blind the participants.

### Outcomes measures

#### Primary outcome measure

- Misbeliefs and attitudes of HP.
  - ○ HC-PAIRS. This self-administered questionnaire was designed to assess attitudes and beliefs of PC physicians on NCLBP. Domenech et al. [44] validated into Spanish the original English version in 2013. After the cross-cultural adaptation, the Spanish version of HC-PAIRS proved to be a reliable, valid and sensitive instrument. The questionnaire consists of 15 statements that suggest a direct link between pain, functional limitation and disability [44]. Each statement is followed by a 7 point Likert scale (1 = strongly disagree, 4 = neither agree nor disagree and 7 = strongly agree). The total score ranges from 1 to 105. Higher scores indicate strong fear-avoidance beliefs on the link between chronic pain and disability [44].

#### Secondary outcome measures

- Fear-avoidance beliefs of HP.
  - ○ Fear Avoidance Beliefs Questionnaire (FABQ). This self-administered questionnaire was developed to measure the beliefs and attitudes of fear-avoidance in patients with LBP on physical and / or occupational activities [45]. The questionnaire is divided into two subscales: FABQ-physical activity and FABQ-work. It consists of 16 sentences on the relationship between pain and physical activity (first 5 items) and work (last 11 items). Each statement is followed by a 6 point Likert scale (0 = strongly disagree and 6 = strongly agree). For both subscales high scores indicate strong fear-avoidance beliefs [45]. This questionnaire has been validated in Spanish. The Spanish version of the FABQ has good internal consistency and reliability. Although it was created to assess fear-avoidance beliefs of patients, this questionnaire has also been used to measure the beliefs of general practitioners and rheumatologists [44, 45].
- Knowledge about NPP.
  - ○ NPP questionnaire. This self-administered questionnaire was developed by Lorimer Moseley in 2003 [46] to assess how an individual conceptualizes pain. An acceptable internal consistency and good test-retest reliability was demonstrated [47]. The original English version was translated into Spanish by the Language Service of the Rovira and Virgili University [48]. The questionnaire consists of 19 statements that refer to the origin and meaning of pain with three possible answers: true, false or doubtful. Each correct answer is scored with 1 point, while incorrect, doubtful or unanswered responses are scored with 0 points [47]. Due to the fact that the questionnaire was designed for patients or HP, the present study omitted the colloquial terms shown in parentheses, using only the terms intended for HP [48].
- Other variables
  - ○ Sociodemographic variables: sex, age, descendency, health discipline and specific training in the vertebral spine.
  - ○ History of LBP.
  - ○ Acceptability and satisfaction of HP about the intervention.
  - ○ Number of consultations to the web site.
  - ○ Number of video views by each participant.

### Web site development and educational material

Once all the information in the QUAL phase has been analyzed and before the QUAN phase begins, the web site and the educational material will be developed. The results of the QUAL phase will be the starting point for the completion of the connection phase, where the educational materials will be developed. The themes and sub-themes that emerge from the QUAL phase will form the basis of the content of the educational material using the NPP. Therefore, the objective of this phase is to respond to the misconceptions about the origin and meaning of pain identified in the QUAL phase using the NPP.

The web site will be created using Drupal content management system. An external researcher from the Higher Polytechnic School of the University of Lleida will take part in this phase. The platform will be built based on the creation of game-based learning scenarios. The application of the elements of games in non-play contexts is called gamification [49, 50]. Its use will provide an innovative and attractive design where HP will be able to work the cognitive processes in a more creative way, thus favoring learning. In addition, it will allow HP to deal with real problems related to the approach of patients who suffer from NCLBP in PC, thus producing changes in their behavior from the stimulus of their motivation [49–53].

The educational material will be presented in different formats, such as explanatory videos and 3D graphic representations, using the chronic NPP. In addition, the authors will use frequent clinical questions about NCLBP patients. Furthermore, HP will be able to contact a chronic pain specialist via email or videoconference [16]. All the videos will be elaborated with the support of the Superior Polytechnic School of the University of Lleida.

### Educational intervention

- Intervention group: This group will have access to our web site where they will find information related to NCLBP. This information will be presented using dynamic explanatory 3D videos. The aim of this intervention is to change and modify misbeliefs and attitudes about NCLBP of physicians and nurses working in PC settings, using a web-based educational tool with the additional result of increasing knowledge on NPP and reducing fear-avoidance beliefs.
- Control group: This group will have access to a video where a group of primary health care professionals explain the content of the CPG for low back spine pathology in adults. This group will also have access to the complete CPG in PDF format.

### Data collection

- QUAL: In order to respond to the QUAL objective of this study, the authors will use semi-structured individual interviews. The first author will be responsible for conducting the interviews. Before the interview, participants will be asked to complete a personal data form for statistical purposes. Interviews will be conducted in Spanish or Catalan (depending on the subject’s mother tongue) and will be recorded in audio with the HP previous consent. In order to promote greater participation, the interviews will be carried out during the participants’ working hours, in authorized spaces within the PC settings, provided by the reference person of the corresponding PC center. The interviewer will have two interview guides, one for the medical staff (see Additional file 1) and one for the nursing personnel (see Additional file 2) built on the knowledge gained from the scientific evidence and the research group’s experience. The questions will be presented in an open format and will be adapted to the HP to which they are addressed. The interviews are meant to last about an hour.
- QUAN: The intervention will last for 2 weeks, and variables will be measured pre- and post-test. The authors based the duration of the intervention on Valenzuela et al. [16] and Keulers et al. [54] studies, because of the similarities in design and characteristics. An external researcher from the Polytechnic University of Lleida will be responsible of encrypting and storing all the data from this study, avoiding any information bias, as well as the maintenance of the web site.
  The first author of this study will meet the study subjects to inform them about the study and deliver the informed consent. Once individuals have signed the consent form, they will be given a password to access the web site. After logging in to the website, HP will need to fill out a personal data form for statistical purposes and will be asked to complete the questionnaires. Once this step has been completed, subjects will see a message on the screen to participate in the control or the intervention group. The control group will be provided a link to have access to an explanatory video based on the CPG for LBP, as well as to the full text of the CPG in PDF format. The intervention group will have access to (will be provided with a personal password) the educational web site from any computer or laptop and at any time, until the end of the trial.
  Both groups will receive two emails during the study (one two days before the end of the study and the second on the last day) to remind them to complete the questionnaires. Once they complete this last step, they will end their participation in the study and will not longer have access to the web site.

### Data analysis

- QUAL: Before beginning the analysis, the interviews will be transcribed verbatim and reviewed by the authors. Deductive and inductive (free or emerging) coding will be used for data identification and integration, resulting in different meaning categories. Different conceptual themes will be generated through which the educational material will be built for the QUAN phase. The first author of the study will analyze the interviews and a different researcher will participate in the information triangulation to ensure the validity and reliability of the material [55, 56]. Subsequently, all the authors will participate in the data interpretation phase and the educational tool development. The Atlas.ti software will be used for the coding process and the information handling.
- QUAN: For statistical purposes the authors will use the SPSS software. For the descriptive analysis, continuous variables will be expressed with the mean and the standard deviation if their distribution is normal, or with the median and the interquartile range, if they do not follow this distribution. Absolute and relative frequency will be used for categorical variables. The t-Student test will be used for the comparison between the continuous variables and the Chi square distribution for the categorical variables. A non-parametric test will be used for the bivariate analysis if a normal distribution cannot be assumed. An alpha error of 0.05 (5%) will be assumed in the statistical inference.

## Discussion

Our research group is studying the treatment of NCLBP from a multidisciplinary approach using techniques included in muscular reeducation and biopsychosocial health education, in the context of PC. For its implementation, our group uses mixed methodologies such as the one described in this study.

The present project aims to give continuity to the study of Valenzuela et al. [35], whose unpublished results suggest that patients’ misbeliefs about NCLBP are caused and / or fed by HP’ fear-avoidance beliefs. These results raise the need to deepen on HP’ beliefs and design effective educational interventions to change cognitive factors in relation to the origin and meaning of chronic pain.

In this context, this study will contribute to the identification of HP’ beliefs about the origin and meaning of chronic pain and its consequences in their attitudes towards the treatment of NCLBP in PC, with the aim of developing a web-based educational tool using different educational formats and gamification techniques.

The results expected in the QUAL phase will provide a better understanding of the misconceptions about the origin and meaning of chronic pain hold by HP in PC, which in turn influence the patients’ beliefs and the clinical advice provided for the management of NCLBP in PC. On the other hand, in the QUAN phase the authors expect that the educational intervention will be effective in the reduction of beliefs and attitudes of fear-avoidance of HP in PC. A better knowledge on the NPP and the reduction of misbeliefs will have a positive impact on the clinical approach of patients suffering from NCLBP, promoting a greater degree of functionality in these patients.

Because NCLBP represents a major health and economic problem for health systems, it is considered necessary the training of medical and nursing staff of PC to improve their efficiency in the management of chronic pain. A HP capable of explaining what the pain is and detecting misbeliefs, including those of fear-avoidance, will allow a better approach to patients suffering from NCLBP, reconceptualizing their beliefs about the origin and meaning of pain and preventing or reducing disability. From the point of view of public health, and given that patients with chronic diseases are the ones that consume more resources, an early detection of misbeliefs in patients with acute low back pain will favor an approach aimed at avoiding the process of chronification caused by the influence of psychosocial factors. This will lead to an improvement in the quality of life of these patients, in addition to a possible reduction in the consumption of drugs as well as in the frequency and / or duration of their time off work.

### Possible limitations

In the elaboration process of the current research protocol, we have detected possible limitations that may discredit the validity and reliability of the results and which are detailed below:

As regards the QUAN phase, one of the possible limitations is that the HP will be aware about the group to which they are assigned and, consequently, the study will be blind only for researchers. Another limitation may be a smaller sample size than expected. A smaller than expected final sample size increases the likelihood of a type II error and limits the internal validity of the study, leading to inconclusive results. However, our results are limited to the HP of the PC centers and should be replicated in randomized control trials with larger sample sizes. A last limitation in this phase could be due to the use of the NPP questionnaire, due to the fact that although it offers valid and reliable results for patients with chronic pain, its psychometric properties have not been investigated in HP [47, 48, 57].

Finally, other limitations may arise from the use of new technologies. In the present project, the educational intervention is based on a web site through which the educational material will be expressed in different formats, such as explanatory videos and 3D images. However, other alternatives and / or formats may have a greater degree of acceptance by the HP in the experimental group [16, 58].

## Additional files

Additional file 1: Interview guide (physicians). The interview guide used for the medical staff (DOCX 16 kb)

Additional file 2: Interview guide (nursing). The interview guide used for the nursing personnel (DOCX 17 kb)

## Abbreviations

CLBP: Chronic Low Back Pain
CPG: Clinical Practice Guidelines
FABQ: Fear Avoidance Beliefs Questionnaire
HC-PAIRS: Health Care Providers Pain and Impairment Relationship Scale
HP: Health Professionals
IASP: International Association of the Study of Pain
ICT: Information and Communication Technologies
LBP: Low Back Pain
NCLBP: Non-specific Chronic Low Back Pain
NPP: Neurophysiology Pain
PC: Primary Care
QUAL: Qualitative
QUAN: Quantitative
