# Supplementary material for: The influence of an educational internet-based intervention in the beliefs and attitudes of primary care professionals on non-specific chronic low back pain: study protocol of a mixed methods approach
Source: BMC Fam Pract. 2019 Feb 21;20:31. doi: 10.1186/s12875-019-0919-6 (PMC6383219; doi:10.1186/s12875-019-0919-6)
Supplement: Supplementary file 1 — Interview guide (physicians). The interview guide used for the medical staff (DOCX 16 kb) [file 12875_2019_919_MOESM1_ESM.docx]

**INTERVIEW GUIDE (PHYSICIANS)**

1. Have you ever suffered or are you currently suffering from low back pain?

(If the answer is yes)

- 1. How did you deal with your pain?
  2. What restrictions did the pain cause you, if any?

1. To your understanding, what is low back pain? What does low back pain mean to you?
2. Which do you think is the cause of NCLBP?
3. Why do you think that the pain ends up chronifying in patients?
4. Which are the diagnostic criteria you use in NCLBP?
5. Which imaging tests do you use most frequently to confirm your diagnosis?
   1. When you demand an imaging test, what results do you expect?
   2. In patients with NCLBP, does your treatment change in the case of hernias or disc protrusions?
      1. If the answer is yes, how does the treatment change?
6. Do you know the alarm standards criteria for NCLBP?
   1. If the answer is yes, do you use them to justify imaging test prescription or referral of the patient to a specialist?
   2. In general terms, do you think they are used in clinical practice by Primary health care professionals?
7. Which is the preference treatment in patients’ who suffer from NCLBP?
   1. In terms of drugs used most commonly, are they effective in pain release in patients with NCLBP?
   2. Which are the drugs you usually prescribe to release pain?
8. What do you think about surgery for NCLBP patients?
   1. What results are obtained in relation to pain release in patients who undergo surgery?
   2. Do you recommend it to your patients?
9. Which are the reasons why you would refer a patient with NCLBP to a specialist (traumatology, rehabilitation service, and /or pain unit)?
10. In your usual practice, do you follow ICS’s CPG for the management of patients with low back pain?
    1. Are you familiar with other CPG to manage NCLBP? Do you use them? Which one?
11. When you diagnose someone with NCLBP, what information do you offer him or her about this condition?
    1. How much time do you spend to provide necessary explanations to and answering questions from your NCLBP patients?
12. What impact do you think that movement and / or physical activity have on pain?
13. What influence has the patient´s mood on the intensity of his pain?
14. What recommendations do you give to your patients with NCLBP?
15. As a healthcare professional, do you like treating patients with NCLBP or would you rather have another professional take care of the patient?
    1. What do you think about your patients’ pain?
16. Do you consider your knowledge about NCLBP enough to treat patients who suffer from it?
17. What would you like to know about NCLBP?
