# Supplementary material for: The influence of an educational internet-based intervention in the beliefs and attitudes of primary care professionals on non-specific chronic low back pain: study protocol of a mixed methods approach
Source: BMC Fam Pract. 2019 Feb 21;20:31. doi: 10.1186/s12875-019-0919-6 (PMC6383219; doi:10.1186/s12875-019-0919-6)
Supplement: Supplementary file 2 — Interview guide (nursing). The interview guide used for the nursing personnel (DOCX 17 kb) [file 12875_2019_919_MOESM2_ESM.docx]

**INTERVIEW GUIDE (NURSING)**

1. Have you ever suffered or are you currently suffering from low back pain?
   1. (If yes) How did you deal with your pain?
   2. What restrictions did the pain cause you?
2. To your understanding, what is low back pain? What does low back pain mean to you?
3. What do you think is the cause of NCLBP?
4. Why do you think that the pain ends up chronifying in patients?
5. What is your opinion towards the diagnosis of NCLBP?
6. Do you consider imaging tests useful and/or necessary to diagnose NCLBP?
7. In your opinion, which are the expectations about the medical interview of NCLBP patients?
8. What role does nursing play in the approach of patients with NCLBP?
9. What do patients associate their pain with?
10. Do you know the alarm standards / alert criteria for NCLBP?
11. What would you change with regard to the NCLBP treatments that are prescribed?
12. What is the patient’s response in relation to drug prescription to reduce pain?
13. What do you think about surgery in NCLBP treatment?
    1. Which is the patient´s reaction towards the possibility of been treated with surgery?
    2. Do you recommend it to your patients?
14. Do you think that patients are satisfied with the attention received by the specialized health teams (traumatology, rehabilitation service and/or pain unit)?
15. In your usual practice, do you follow ICS’s CPG to approach patients who suffer from NCLBP?
    1. Are you familiar with other CPG to approach NCLBP? Do you use them?
16. What information do you give to your NCLBP patients?
    1. How much time do you spend to provide necessary explanations to and answering questions from your NCLBP patients?
    2. What questions do patients with NCLBP have most frequently?
17. What impact do you think that movement and / or physical activity have on pain?
18. What kind of influence do you think patient's mood has on the intensity of their pain?
19. What recommendations do you give to patients with NCLBP?
20. As a healthcare professional, do you like treating patients with NCLBP or would you rather have another professional take care of the patient?
    1. What do you think about your patients’ pain?
21. Do you consider your knowledge about NCLBP enough to treat patients who suffer from it?
22. What would you like to know about NCLBP?
